# Proteogenomic landscape of uterine leiomyomas from hereditary leiomyomatosis and renal cell cancer patients

**DOI:** 10.1038/s41598-021-88585-x

**Published:** 2021-04-30

**Authors:** Nicholas W. Bateman, Christopher M. Tarney, Tamara Abulez, Anthony R. Soltis, Ming Zhou, Kelly Conrads, Tracy Litzi, Julie Oliver, Brian Hood, Paul Driggers, Coralie Viollet, Clifton Dalgard, Matthew Wilkerson, William Catherino, Chad A. Hamilton, Kathleen M. Darcy, Yovanni Casablanca, Ayman Al-Hendy, James Segars, Thomas P. Conrads, G. Larry Maxwell

**Affiliations:** 1 Gynecologic Cancer Center of Excellence, Department of Gynecologic Surgery and Obstetrics, Uniformed Services University of the Health Sciences and Walter Reed National Military Medical Center, 8901 Wisconsin Avenue, Bethesda, MD 20889 USA; 2 The John P. Murtha Cancer Center, Uniformed Services University of the Health Sciences and Walter Reed National Military Medical Center, 8901 Wisconsin Avenue, Bethesda, MD 20889 USA; 3 Henry M. Jackson Foundation for the Advancement of Military Medicine, Inc, 6720A Rockledge Dr., Suite 100, Bethesda, MD 20817 USA; 4 The American Genome Center, Department of Anatomy, Physiology, and Genetics, Collaborative Health Initiative Research Program, Uniformed Services University of the Health Sciences, 4301 Jones Bridge Road, Bethesda, MD 20814 USA; 5 Women’s Health Integrated Research Center, Women’s Service Line, Inova Health System, 3300 Gallows Rd, Falls Church, VA 22042 USA; 6 Johns Hopkins University School of Medicine, Baltimore, MD USA; 7 Department of Gynecologic Surgery and Obstetrics, Uniformed Services University of the Health Sciences, Bethesda, MD USA; 8 University of Illinois College of Medicine, Chicago, IL 60612 USA; 9 3289 Woodburn Rd, Suite 375, Annandale, VA 22003 USA

**Keywords:** Endometrial cancer, Proteomic analysis

## Abstract

Pathogenic mutations in fumarate hydratase (*FH*) drive hereditary leiomyomatosis and renal cell cancer (HLRCC) and increase the risk of developing uterine leiomyomas (ULMs). An integrated proteogenomic analysis of ULMs from HLRCC (n = 16; *FH*-mutation confirmed) and non-syndromic (NS) patients (n = 12) identified a significantly higher protein:transcript correlation in HLRCC (R = 0.35) vs. NS ULMs (R = 0.242, MWU p = 0.0015). Co-altered proteins and transcripts (228) included antioxidant response element (ARE) target genes, such as thioredoxin reductase 1 (*TXNRD1*), and correlated with activation of NRF2-mediated oxidative stress response signaling in HLRCC ULMs. We confirm 185 transcripts previously described as altered between HLRCC and NS ULMs, 51 co-altered at the protein level and several elevated in HLRCC ULMs are involved in regulating cellular metabolism and glycolysis signaling. Furthermore, 367 S-(2-succino)cysteine peptides were identified in HLRCC ULMs, of which sixty were significantly elevated in HLRCC vs. NS ULMs (LogFC = 1.86, MWU p < 0.0001). These results confirm and define novel proteogenomic alterations in uterine leiomyoma tissues collected from HLRCC patients and underscore conserved molecular alterations correlating with inactivation of the *FH* tumor suppressor gene.

## Introduction

Uterine leiomyomas (ULMs) are the most common benign gynecologic tumor occurring in 70–80% of women in the United States by the end of their reproductive years^1,2^. Approximately 15–30% of women with ULMs will have significant symptoms including abnormal uterine bleeding, pelvic pain, and infertility^3,4^. The economic impact of ULMs is profound with recent estimates related to the cost of medical care being as high as $34 billion annually in the United States^5^. Despite the prevalence and burden of ULMs, there is a limited understanding of the molecular underpinnings and pathogenesis of this disease.

Examining hereditary syndromes associated with an increased risk of developing ULMs provides a unique opportunity to evaluate the underlying molecular causes of ULMs. Hereditary leiomyomatosis and renal cell cancer (HLRCC) is an autosomal-dominant disease that is associated with cutaneous leiomyomas, uterine leiomyomas, leiomyosarcoma, and papillary renal cell cancer^6^. This syndrome is caused by deleterious mutations in the tumor suppressor gene that encodes fumarate hydratase (*FH*), an enzyme that converts fumarate to malate and is integral to the Krebs cycle and cellular metabolism^7^.

Molecular investigations of HLRCC may provide insight into the etiology of ULMs and tumorigenesis that includes deleterious mutations of *FH* as this alteration results in mitochondrial dysfunction and energy depletion, which can promote DNA damage as a product of elevated free radicals and activation of hypoxia-responsive signal transduction pathways that can further lead to angiogenesis and growth factor production^8,9^. To improve our understanding of the molecular etiology of ULMs, we conducted a comprehensive proteogenomic analysis of ULM tissue specimens from a cohort of HLRCC patients and those with non-syndromic (NS) ULMs. Our integrative analysis of whole genome sequencing (WGS), RNA-sequencing (RNA-seq) as well as quantitative mass spectrometry (MS)-based proteomics identified putative novel molecular drivers associated with disease pathogenesis in HLRCC patients. Integrated analyses of these data provide a comprehensive view into conserved proteogenomic alterations underlying the molecular etiology of uterine leiomyoma development in HLRCC patients as well as in tumors harboring deleterious mutations in *FH*.

## Results

### Integrated Proteogenomic Analyses of HLRCC and Non-Syndromic ULMs

**Figure 1:**
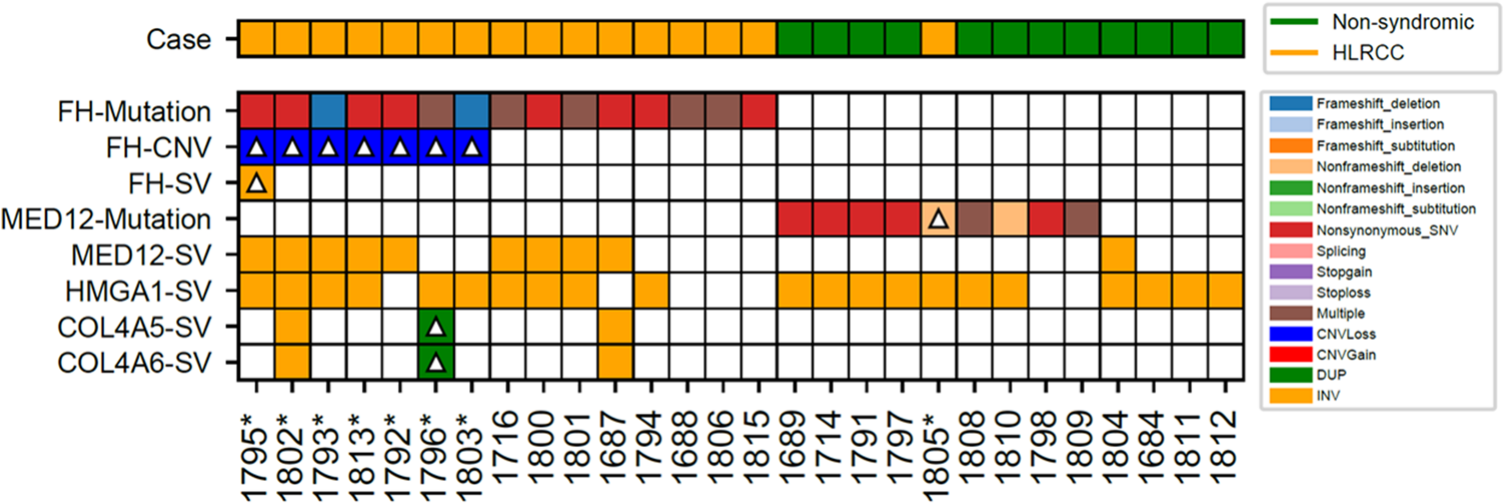
Mutation, Copy Number Variation and Structural Variant (SV) Analysis of Fumarate Hydratase (*FH*) and in Genes Associated with Uterine Leiomyoma Development. Heatmap details single nucleotide variants (SNVs), insertion/ deletion, copy number variation (CNV) events as well as cases harboring multiple variants in *FH* or uterine leiomyoma driver genes; Mediator Complex Subunit 12 (*MED12*), High Mobility Group AT-Hook 1 (*HMGA1*), Collagen Type IV Alpha 5 Chain (*COL4A5*) and collagen type IV alpha 6 chain (*COL4A6*)^10^. Mutations in *FH* and *MED12* were directly called from ULM whole genome sequencing data aside from patients designated with a “*” that included sequencing of a matched myometrium sample supporting somatic mutation analysis of this patient subset; mutations designated with a triangle in these select cases represent somatic alterations identified in ULMs alone. Somatic mutation analyses of n7 HLRCC patients revealed several harbored germline mutations in *FH* as well as copy number loss of chromosome 1 regions encoding *FH*; see Supplemental Table 2 for details.

**Figure 2:**
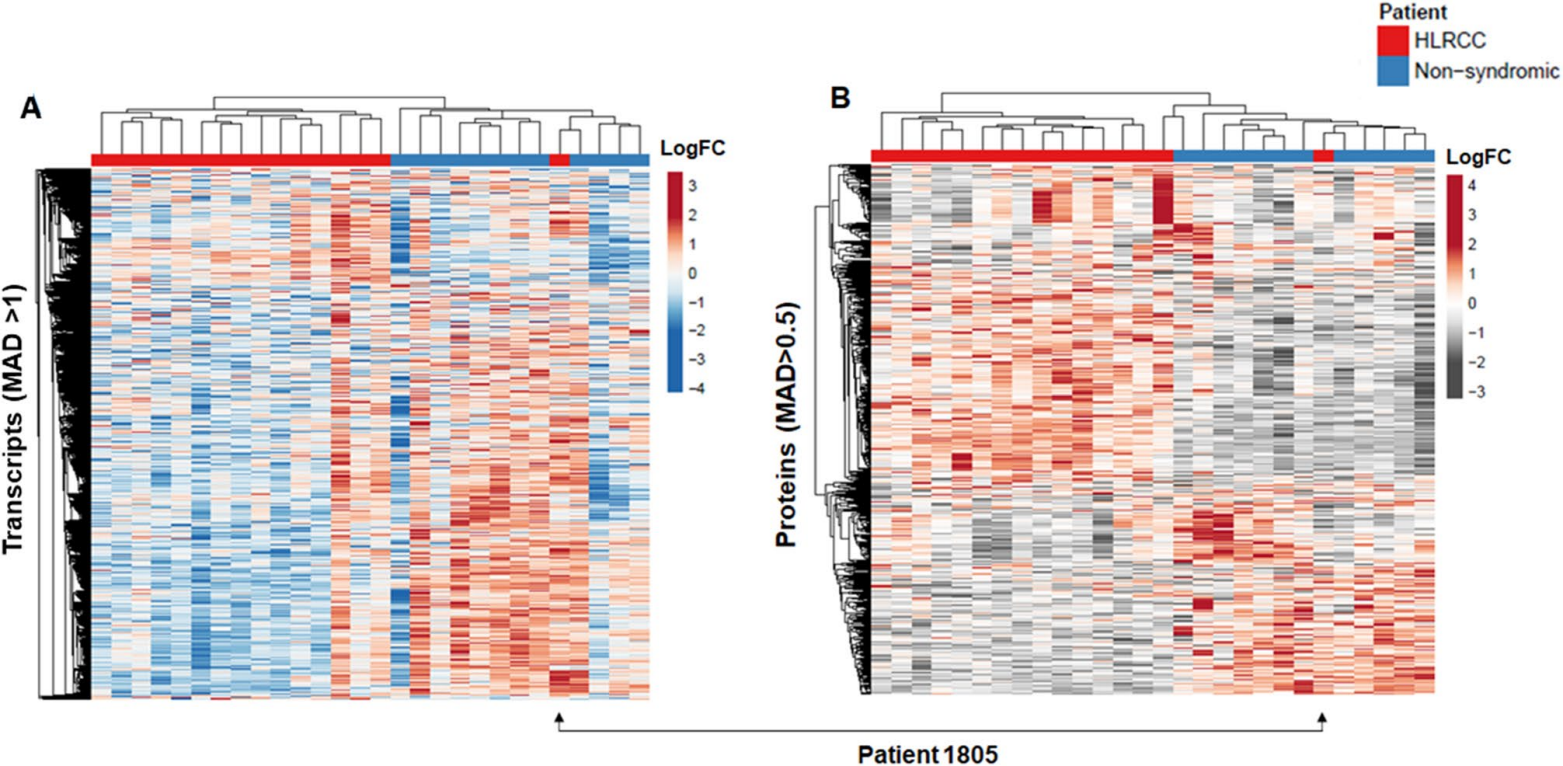
Unsupervised cluster analysis of protein and transcript alterations in uterine leiomyomas (ULMs) from Hereditary Leiomyomatosis and Renal Cell Cancer (HLRCC) and non-syndromic (NS) patients. Heatmaps detail (**A**) transcripts and (**B**) proteins quantified exhibiting a median absolute deviation (MAD) in total RNA-seq (MAD > 1) or TMT-10 quantitative proteomics (MAD > 0.5) data; clusters organized by correlation and average linkage (ClustVis). Plots reveal one HLRCC patient, designated as 1805, clustering as a non-syndromic ULM.

We conducted a comprehensive proteogenomic analysis employing WGS, RNA-seq, and quantitative MS-based proteomics of fresh-frozen ULM specimens from NS women (n = 12) and from women (n = 16) with HLRCC based on genetic counseling and family history assessments (Supplemental Table 1). Analyses of mutational status in fumarate hydratase (*FH)* as well as Mediator Complex Subunit 12 (*MED12*) confirmed that all suspected HLRCC patients exhibited a hotspot mutation or insertion/deletion (indel) event in the gene encoding *FH,* except patient 1805, in which a deletion (c.124_135del:p.42_45del) was found in exon2 of *MED12* (Fig. 1, Supplemental Table 2). The lack of an *FH* mutation in patient 1805 is consistent with this case clustering with non-syndromic ULMs based on transcript and protein-level evidence (Fig. 2A,B, discussed below) and suggests this case is not an HLRCC patient. Non-syndromic patient samples largely exhibited deleterious mutations in *MED12* as well as inversion variants in additional gene targets identified as commonly altered in ULMs^10^, namely high mobility group AT-hook 1 (*HMGA1*). Structural variants in *MED12* and *HMGA1* were also common in HLRCC patients with a subset (cases 1796, 1802 and 1687) exhibiting duplications or inversions in Collagen Type IV Alpha 5 Chain (*COL4A5*) and collagen type IV alpha 6 chain (*COL4A6*). Analyses of WGS data from matched myometrium for 7 of the 16 HLRCC patients identified a somatic mutation frequency of 0.587 ± 0.884/Mb in patient ULMs, a result that is consistent with tumors that have lower mutational burdens, such as Rhabdoid tumor and Ewing sarcoma^11^.

RNA sequencing quantified > 20 K (Supplemental Table 3) transcripts, among which 3,411 were co-quantified at the protein level. Proteins quantified without transcript-level evidence were largely predicted to be localized to the extracellular space, including multiple IgG isoforms and were enriched for cellular signaling pathways such as complement activation and immune regulation consistent with being immune cell or blood serum-derived (Supplemental Table 4). Unsupervised analyses of 991 variably abundant transcripts (median absolute deviation (MAD) > 1) stratified HLRCC and NS patients (Fig. 2A). Notably, HLRCC patient 1805 clustered with non-syndromic patients in this analysis.

Global quantitative proteomic analyses using a multiplexed tandem mass tag (TMT-10) workflow quantified 3,510 proteins across all patient samples (Supplemental Table 5). These analyses included a subset of replicate ULMs collected from seven HLRCC patients where the majority, n = 5 replicate tumors, exhibited high correlation of protein abundances, Spearman R = 0.6 ± 0.15. However, the remaining n = 2 subset exhibited lower correlations, Spearman R = 0.21 ± 0.05 (Supplemental Table 6); suggesting most tumors from the same patient exhibit more conserved protein abundances, although we do observe higher heterogeneity in some instances which represents a limitation of comparative analyses of single patient tumors. Unsupervised analyses of 736 variably abundant proteins (MAD > 0.5) stratified HLRCC and NS patients (Fig. 2B). We again note that HLRCC patient 1805 clustered with NS patients based on unsupervised analysis of protein alterations. We further assessed the abundance of FH and found this protein was significantly decreased in HLRCC patients (median difference HLRCC versus NS ULMs, -1.39, MWU p < 0.0001 (Supplemental Table 5). Loss of FH protein abundance in HLRCC patients is consistent with the often protein-destabilizing, autosomal dominant mutations that are common in HLRCC^12^.

### Integrated analyses of transcriptome and proteome expression

**Figure 3:**
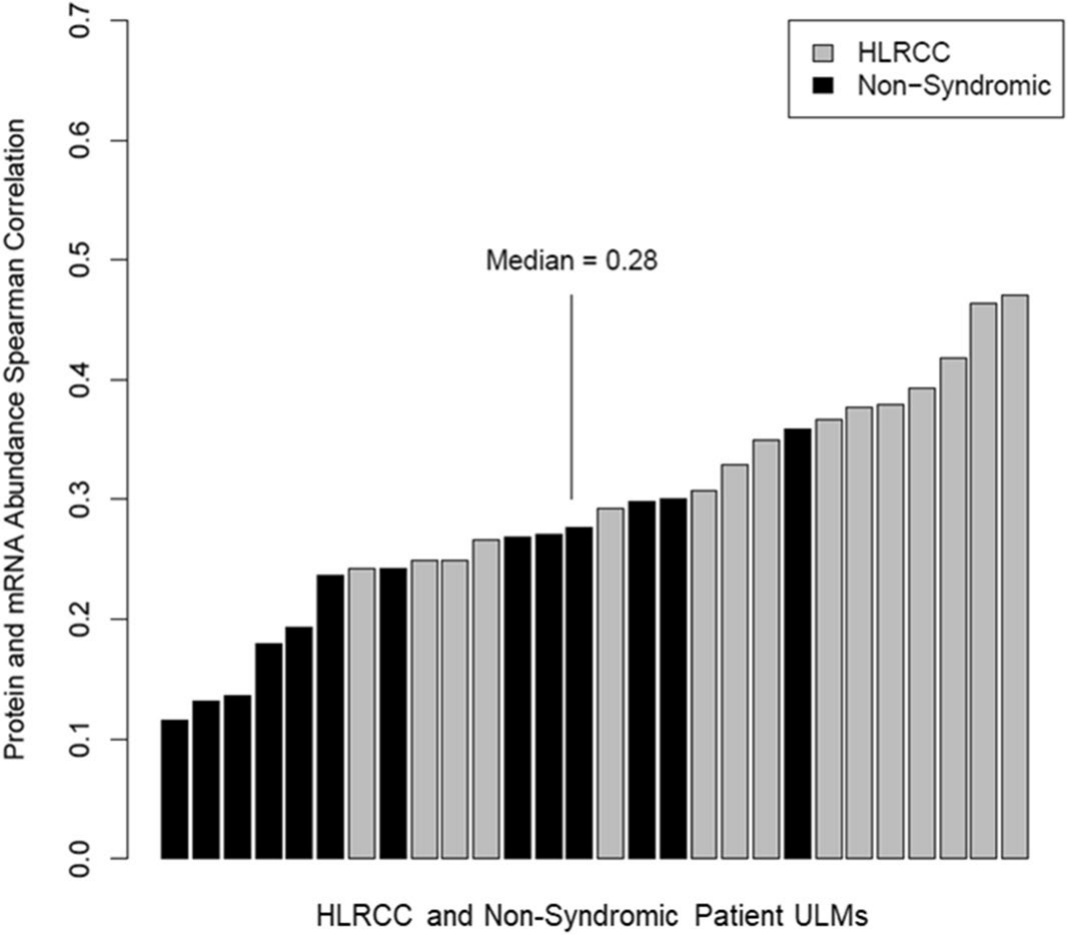
Correlation of protein and transcript abundance in ULMs from Hereditary Leiomyomatosis and Renal Cell Cancer (HLRCC) and non-syndromic (NS) patients. Bar plot details Spearman Rho correlations calculated for each ULM analyses based on 3411 co-quantified proteins and transcripts. Median Spearman Rho for HLRCC, R = 0.35 was significantly higher than for non-syndromic ULMs, R = 0.242, Mann Whitney U p = 0.0015.

Integrated analyses of co-quantified proteins and transcripts in individual ULMs revealed a median correlation of Spearman Rho = 0.28 across all ULMs (Fig. 3). We further observed that HLRCC tumors exhibited significantly higher correlation of co-quantified protein and transcript abundances (Spearman R = 0.35) versus NS ULMs (Spearman R = 0.242, MWU p = 0.0015) (Supplemental Table 7). As the total number of co-quantified proteins and transcripts is less than that reported by similar correlation analyses, such as in endometrial cancers (EC), e.g. Dou et al.^13^, who report correlation analyses of > 10,000 proteins and transcripts, we investigated whether this reduced feature set may represent an analytical bias for our analyses. We performed correlation analyses of a subset of 3292 transcripts and proteins from those co-quantified in ULMs across n = 101 EC patient tumors; these subset features represent proteins and gene names that matched supplemental data in Dou et al., and we observed the median Spearman correlation for this feature subset was R = 0.52 ± 0.11. To confirm performance of our correlation method, we assessed correlation for all proteins and transcript co-quantified across n > 100 patient tumor samples, 10,986 pairs, and observed a median R = 0.49 ± 0.1 which is consistent with the median R = 0.48 reported by Dou Y et al. Although ULMs and endometrial cancers represent distinct neoplasms, this comparative analyses suggests that our co-quantified feature subset is unlikely to bias our correlation analyses. Notably, a recent comparison of protein and transcript abundances measured in tumors and normal adjacent tissues from > 100 lung cancer patients has shown that median transcript and protein abundance correlations trend as lower in normal in comparison to tumor tissues^14^. As ULMs are benign neoplasms, the lower overall median correlation of protein and transcript abundances that we observe in relation to malignant tumors is consistent with the benign status of ULMs. However, our observation that ULMs from HLRCC exhibit significantly higher protein and transcript correlations than NS ULMs suggest perhaps HLRCC ULMs may share this characteristic with more aggressive tumors. We further investigated proteins and transcripts exhibiting differential correlation abundance trends in HLRCC vs NS ULMs (Supplemental Table 8). We identified 347 pairs that exhibit highly variable (MAD > 0.5) correlation trends in HLRCC vs NS ULMs. Pathway analyses of those positively correlated (111, Spearman Rho > 0.6) in HLRCC ULMs revealed enrichment of pathways correlating with metabolism, namely the 6-Phosphofructo-2-Kinase/Fructose-2,6-Biphosphatase 4 (PFKFB4) pathways as well as aerobic respiration, (tricarboxylic acid cycle II). Highly correlated pairs in NS ULMS (46, Spearman Rho > 0.6) were enriched for mitochondrial dysfunction as well as VEGF signaling (Supplemental Table 8).

**Figure 4:**
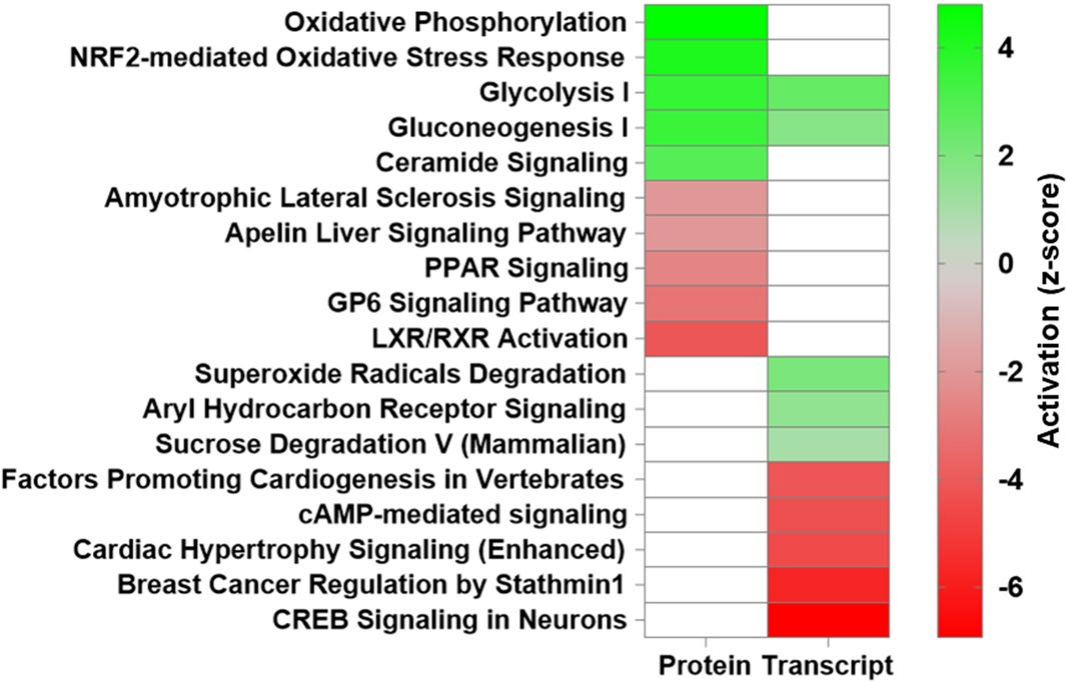
Canonical pathways enriched by proteins and transcripts significantly altered in ULMs from Hereditary Leiomyomatosis and Renal Cell Cancer (HLRCC) and non-syndromic (NS) patients. Heatmap reflects the top 10 canonical pathways predicted as activated (positive z-score) or inhibited (negative z-score) based on proteins and transcripts significantly altered between HLRCC and non-syndromic ULMs (LIMMA adjusted p < 0.01). Pathways colored white not among top pathways predicted to be activated or inhibited in protein or transcript level data. Functional enrichment and causal analyses performed using Ingenuity Pathway Analysis^15^.

**Figure 5:**
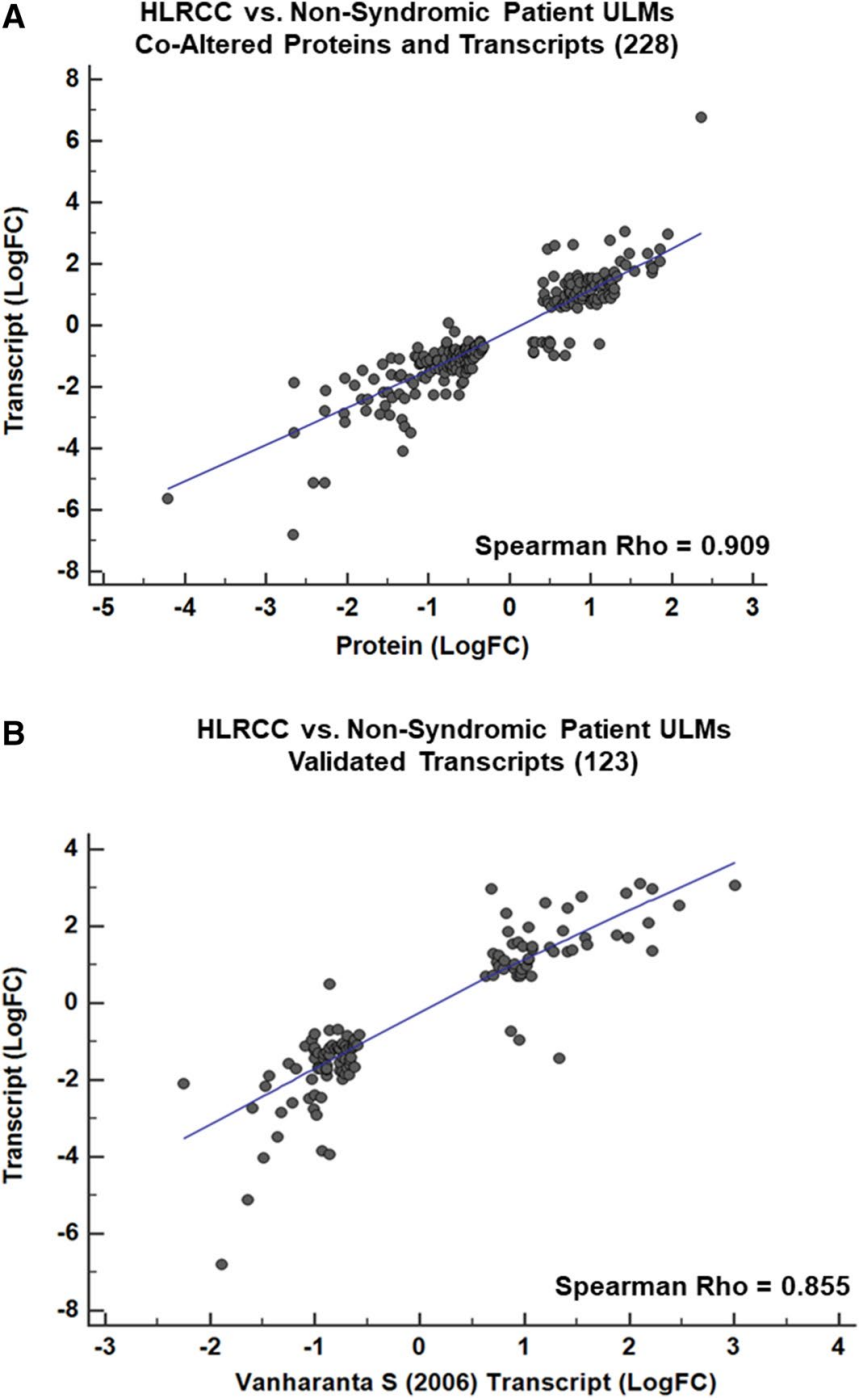
Correlation plot of co-altered proteins, transcripts and validated transcript alterations in Hereditary Leiomyomatosis and Renal Cell Cancer (HLRCC) and non-syndromic (NS) patients. (**A**) Correlation plot reflects 228 co-altered proteins and transcripts significantly altered between HLRCC and NS ULMs (Spearman Rho = 0.909, MWU p < 0.0001). (**B**) Correlation plot reflects 123 transcripts co-altered in our dataset and Vanharanta et al.^20^ (Spearman Rho = 0.855, MWU p < 0.0001).

**Figure 6:**
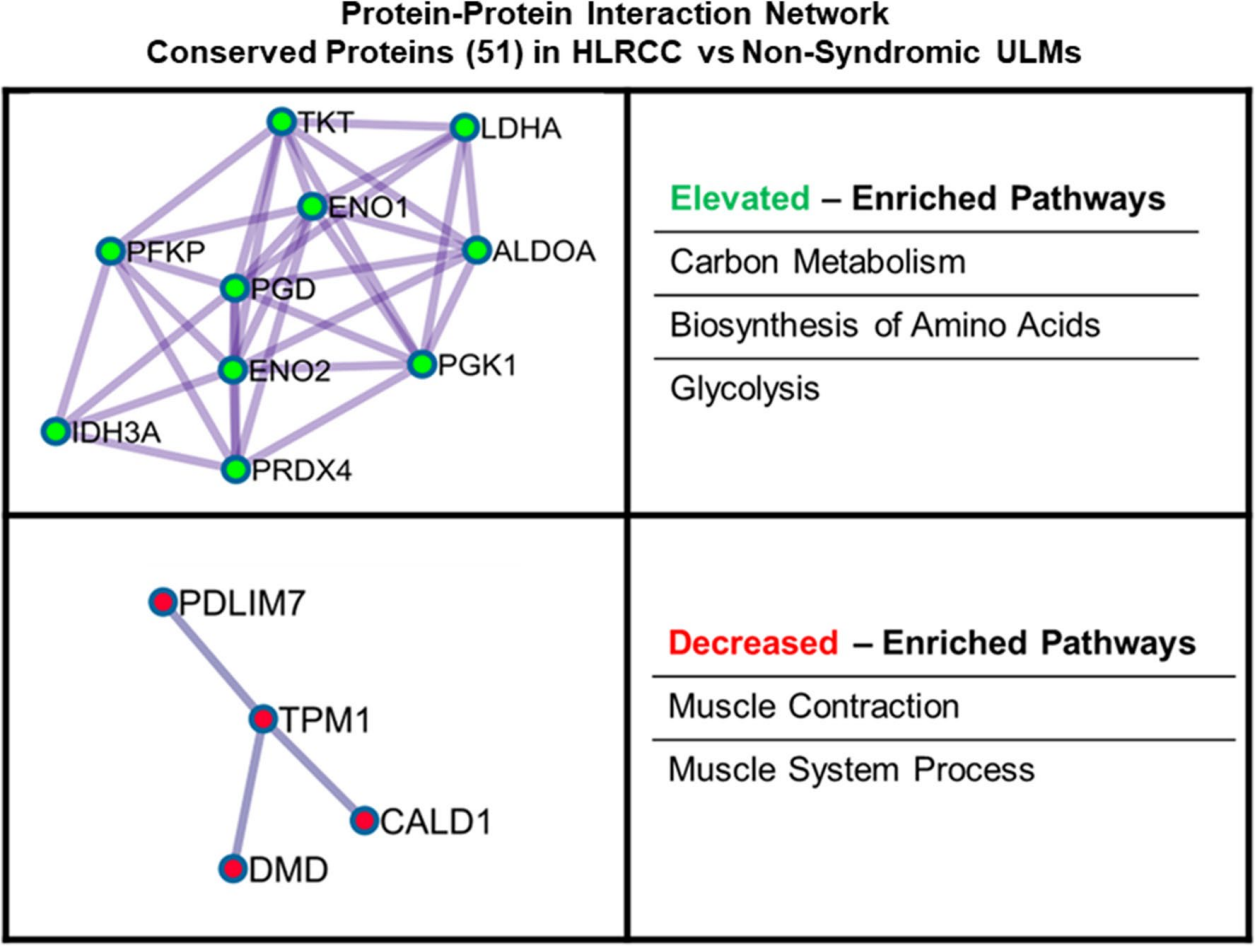
Protein interaction network enriched from validated transcripts confirmed at the protein-level as altered between HLRCC versus non-syndromic (NS) ULMs. Validated transcripts confirmed at the protein level (51 total) were analyzed using MetaScape^51^ and resulting protein–protein interaction networks enriched by proteins elevated (highlighted in green) or decreased (heighted in red) in HLRCC vs NS ULMs were assembled using Cytoscape^52^. The top pathways enriched by network proteins are also noted.

Pathway analyses of proteins (Supplemental Table 9) or transcripts (Supplemental Table 10) significantly (LIMMA adjusted p-value < 0.01) co-altered between confirmed HLRCC (n = 15) and NS ULMs (n = 13) revealed little overlap between top canonical signaling pathways predicted to be activated or inhibited in these tumors (Fig. 4, Supplemental Table 11). Co-altered pathways included glycolysis and gluconeogenesis signaling predicted to be functionally activated^15^ in HLRCC versus NS ULMs based on both protein and transcript alterations (Fig. 4, Supplemental Table 11). As our patient cohort is racially diverse (for example the majority of HLRCC tumors are collected from European-American (EA) women whereas the NS tumors were from African-American (AA) women, Supplemental Table 1), we sought to identify the extent to which transcripts associated with central metabolism could be biased by patient race by comparison with a recently described multi-omic analyses of NS ULMs collected from EA (n = 8) and AA (n = 5) women^16^. We assessed 16 transcripts associated with glycolysis and gluconeogenesis signaling in our dataset and found enolase 2 among this subset as significantly altered (ENO2, LogFC + 1.02 in HLRCC vs NS ULMs) in ULMs from EA vs AA women (LogFC − 0.64, LIMMA P < 0.05), suggesting alterations observed in the majority of these cases are not impacted by patient race. We further identify an additional 16 pathways co-enriched based on transcript and protein alterations, with a subset predicted to exhibit comparable activation trends, such as sucrose degradation V (mammalian) and superoxide radicals degradation, but others, such as calcium and thrombin signaling as well as unfolded protein response are predicted as activated at the protein level, but inhibited based on transcript level abundance. Notably, we observed activation of the “NRF2-mediated oxidative stress response” in HLRCC versus NS patients as a top altered pathway based on protein-level alterations that was also significantly enriched at the transcript level (Supplemental Table 11). We further investigated the 35 transcripts altered between HLRCC and NS tumors enriched for the NRF2-mediated oxidative stress response pathway for association with race in NS tumors and none were significantly altered in ULMs from EA vs AA women from George et al.^16^; these were also not associated with racial status in our study (data not shown). As the NRF2 antioxidant response pathway and downstream transcriptional targets have been implicated as key drivers of tumorigenesis in *FH* mutant tumors^17^, we investigated the prevalence of co-altered transcripts and proteins harboring antioxidant response element (ARE) promoter binding motifs. This comparison revealed 228 significantly co-altered protein:transcript pairs between HLRCC and NS ULMs that exhibited high quantitative correlation (Spearman Rho = 0.909, p < 0.0001) (Fig. 5A, Supplemental Table 12); 17 of which harbor high-confidence ARE promoter binding motifs^18^, with the majority being significantly elevated in HLRCC vs. NS ULMs (Table 1, Supplemental Table 13). Among this list, we observe both ferritin light chain (*FTL*) and ferritin heavy chain (*FTH*) (Table 1) to be significantly elevated at the protein and transcript level. Both *FTL* and *FTH* are NRF2 target genes that have been described as being increased in *FH* mutant cells and to further directly mediate a pro-proliferative signal^19^. Pathway analyses of subset, co-altered protein:transcript pairs that further exhibit identical protein and transcript abundance trends (214total) showed activation of pathways regulating proliferation of tumor cells as well as glycolysis signaling and inhibition of pathways regulating oxidative stress as well as cell spreading in HLRCC versus NS ULMs (Table 2, Supplemental Table 14). These co-altered candidates include multiple putative drug targets elevated in HLRCC versus NS patients, such as arsenic trioxide targeting the NRF2-target gene thioredoxin reductase (*TXNRD1*), as well as additional metabolic targets including the cyclic heptapeptide CAP-232 (TLN-232) targeting the glycolytic enzyme complex resident protein pyruvate kinase M2 (Table 3). We also compared transcript alterations in our study with a previously published cDNA microarray analysis of *FH* mutant (n = 7) versus *FH* wildtype (n = 15) ULMs (Vanharanta et al.^20^). Of 360 altered probesets reported by Vanharanta S mapping to 234 genes in our dataset, we find 185 are significantly co-altered (LIMMA P < 0.05) between HLRCC vs NS ULMs including a subset of 123 significant (LIMMA adjusted p < 0.01) alterations that exhibit high quantitative correlation between these independent cohorts (Spearman Rho = 0.855, p < 0.0001) (Fig. 5B, Supplemental Table 15). The remaining 48 transcript alterations described by Vanharanta S et al. not significantly co-altered (LIMMA p > 0.05) in our dataset were quantitatively correlated with HLRCC vs NS ULMs (Spearman Rho = 0.348, p = 0.014). Comparison with companion protein-level data revealed 51 were also significantly co-altered and highly quantitatively correlated at the protein level (Spearman Rho = 0.924, p < 0.001). Among these, ten proteins elevated in HLRCC versus NS harbor ARE binding elements (designated with * in Table 1) and several are known to participate in protein–protein interactions regulating carbon metabolism as well as cellular glycolysis (“Elevated”, Fig. 6). Further, several are decreased in HLRCC cases regulate muscle contraction (“Decreased”, Fig. 6). This subset of 51 transcript:protein pairs validate previously defined alteration trends and represent highly conserved alterations in HLRCC ULMs including multiple candidates containing ARE gene promoter motifs that are likely NRF2 transcriptional targets as well as those regulating key steps in glycolysis, such as alpha-enolase (*ENO1*) and gamma-enolase (*ENO2*), both involved in catalyzing the conversion of 2-phosphoglycerate to phosphoenolpyruvate, as well as phosphoglycerate kinase 1 (*PGK1*) regulating conversion of 1,3-diphosphoglycerate to 3-phosphoglycerate^21^.

**Table 1:** Protein and transcript alterations in HLRCC versus Non-Syndromic ULMs. with antioxidant response element (ARE) promoter binding motifs.

Symbol	Entrez gene name	HLRCC vs. NS ULM	HLRCC vs NS ULM
		(Protein, LogFC)	(Transcript, LogFC)
NQO1*	NAD(P)H dehydrogenase [quinone] 1	3.06	1.42
HMOX1*	Heme oxygenase 1	2.48	0.47
FTL*	Ferritin light chain	2.34	1.70
TXNRD1	Thioredoxin reductase 1, cytoplasmic	2.33	1.47
SQSTM1*	Sequestosome-1	1.59	0.55
FTH1*	Ferritin heavy chain	1.49	1.24
GCLM*	Glutamate–cysteine ligase regulatory subunit	1.35	1.16
PRDX1*	Peroxiredoxin-1	1.35	1.25
SLC2A1	Solute carrier family 2, facilitated glucose transporter member 1	1.32	1.06
ETFB*	Electron transfer flavoprotein subunit beta	0.89	1.24
IDH3A*	Isocitrate dehydrogenase [NAD] subunit alpha, mitochondrial	0.70	1.00
CPB2	Carboxypeptidase B2	0.08	− 0.75
CIRBP*	Cold-inducible RNA-binding protein	− 0.94	− 0.54
TAGLN	Transgelin	− 1.25	− 1.56
ITGA1	Integrin alpha-1	− 1.39	− 0.80
UCHL1	Ubiquitin carboxyl-terminal hydrolase isozyme L1	− 1.73	− 1.23
STMN2	Stathmin-2	− 1.96	− 1.90

Candidates designated with a * exhibit concordant abundance trends between HLRCC and NS ULMs in Vanharanta et al.^20^.

**Table 2:** Pathway analyses of conserved protein and transcript alterations in HLRCC versus Non-Syndromic (NS) ULMs.

Diseases or Biofunctions	Activation z-score
Cell proliferation of tumor cell lines	3.699
Abdominal neoplasm	2.642
Glycolysis of cells	2.567
Growth of tumor	2.52
Cell proliferation of sarcoma cell lines	2.338
Migration of cells	−1.98
Cell spreading of tumor cell lines	−2.019
Shape change of tumor cell lines	−2.222
Cytostasis	−2.237
Quantity of reactive oxygen species	−2.318

Table denotes top 5 functional pathways predicted to be activated (positive z-score) or inhibited (negative z-score) in HLRCC vs NS ULMs (Ingenuity pathway analyses, p-value < 0.05).

**Table 3:** Conserved protein and transcript alterations in HLRCC versus Non-Syndromic (NS) ULMs that are putative drug targets.

Gene ID	Protein name	HLRCC vs. NS ULM	Drug(s)
		(Protein, LogFC)	
TXNRD1	Thioredoxin reductase 1	1.471	Arsenic trioxide/tretinoin
MIF	Macrophage migration inhibitory factor	1.091	Imalumab
CDK6	Cyclin dependent kinase 6	1.027	Palbociclib
PKM	Pyruvate kinase M1/2	0.963	CAP-232
GSR	Glutathione-disulfide reductase	0.9	Carmustine/prednisone
TFRC	Transferrin receptor	0.856	CX-2029
AKR1C3	Aldo–keto reductase family 1 member C3	0.849	ASP9521
SRC	SRC proto-oncogene, non-receptor tyrosine kinase	0.47	Blinatumomab/dasatinib
HMOX1	Heme oxygenase 1	0.469	Tin mesoporphyrin
MAPKAPK2	MAPK activated protein kinase 2	0.411	cdc25C phosphatase (211–221)
GUCY1A2	Guanylate cyclase 1 soluble subunit alpha 2	− 0.489	Nitroprusside
CTPS1	CTP synthase 1	− 0.531	Cytidine triphosphate synthetase inhibitor
TUBB2A	TUBULIN beta 2A class IIa	− 0.653	Epothilone B
DMD	dystrophin	− 0.662	Golodirsen
COL#X#	Multiple collagen Isoforms COL6A1, COL6A3, COL5A1, COL12A1, COL16A1, COL1A1, COL15A1	≤ − 0.586	Collagenase clostridium histolyticum
MARCKS	Myristoylated alanine rich protein kinase C substrate	− 1.185	BIO-11006
CA3	Carbonic anhydrase 3	− 2.035	Hydrochlorothiazide

Table denotes top elevated or decreased alterations in HLRCC vs NS ULMs that are putative drug targets (Ingenuity pathway analyses).

To investigate alterations in HLRCC versus NS ULMs important for survival of *FH* mutant cells, we crossed significantly altered proteins with 340 gene targets previously identified as conferring synthetic lethality in *FH* mutant cell lines when co-silenced^22^. We find that twenty-seven proteins significantly (LIMMA adjusted p < 0.01) altered in HLRCC versus non-syndromic ULMs overlapped with this target list (Supplemental Table 16). The majority of alterations, 18 candidates,were elevated in HLRCC cases and the most abundant included several metabolic regulatory targets, including lactate dehydrogenase (LDHA, + 1.54 LogFC in HLRCC versus NS), glucose transporter 1 (SLC2A1, + 1.06 LogFC), enolase 3 (ENO3, + 0.7 LogFC), succinate dehydrogenase (SDHA, + 0.65 LogFC) as well as proteasome activator complex subunit 1 (PSME1, + 0.67). We further identified 9 proteins that were decreased in HLRCC ULMs including several predicted to be localized to the extracellular matrix, such as fibulin 2 (FBLN2, − 1.35) and laminin subunit alpha 2 (LAMA2, − 1.11) as well as the transcription factor PBX homeobox 1 (PBX1, − 0.66).

### HLRCC ULMs exhibit widespread S-(2-succino)cysteine (2SC) modified peptides

**Figure 7:**
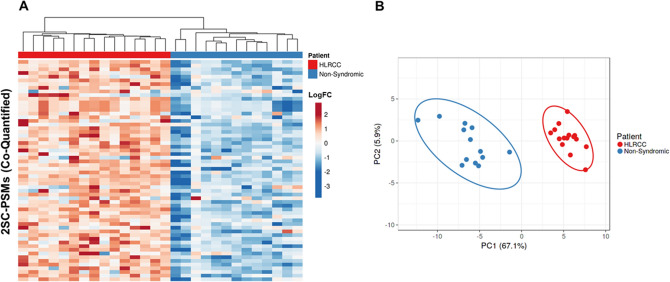
Unsupervised cluster analysis of quantified peptides exhibiting 2-succinyl modified cysteine residues in uterine leiomyomas (ULMs) from Hereditary Leiomyomatosis and Renal Cell Cancer (HLRCC) and non-syndromic (NS) patients. (**A**) Heatmaps detail 60 peptide spectral matches (PSMs) encoding 2-succinyl modified cysteine residues quantified in HLRCC and non-syndromic ULMs; clusters organized by correlation and average linkage (ClustVis). (**B**) Principle component analyses of these 60 2SC-PSMs revealed the abundance of these features can explain 67.1% of the variance (PC1) and 5.9% of the variance (PC2) between these patient cohorts; median difference of 2SC-PSMs = 1.86, MWU p < 0.0001.

The accumulation of cellular fumarate resulting from mutated *FH* can result in succination of cysteinyl-containing peptides, a post-translational modification (PTM) that contributes a static 116.1 Da mass addition to these peptides that is readily identifiable by MS^23^. A targeted search of our proteomics data for this cysteinyl PTM quantified a total of 367 unique 2SC peptides, corresponding to 253 unique protein targets (Supplemental Table 17). We quantified 60 2SC peptides across all patient samples and observed that elevated abundance of these modified peptides was highly specific for HLRCC cases (Fig. 7A), significantly stratifying HLRCC from NS patients (Fig. 7B, principal component 1 and principal component 2 explain 67.1% and 5.9% of the total variance; HLRCC vs NS ULMs = 1.86, MWU p < 0.0001). Several co-quantified protein targets exhibited multiple 2SC peptides and include thioredoxin (TXN), a redox-regulated protein that mediates dithiol-disulfide exchanges and S-nitrosylation of cysteine residues in multiple target proteins and is a direct target of TXNRD1 that we quantified as elevated in HLRCC versus NS ULMs^24,25^. We quantified one such TXN PSM bearing 2SC modification of C73, a residue that is necessary for mediating nitrosylation of an active site cysteine residue in the pro-apoptotic protein caspase 3^25^. We further correlated 2SC peptides with a recently published analyses of 2SC-containing peptides discovered in cell line models of HLRCC^26^ and validate fifty-three 2SC peptides described by this group including eighteen also previously identified in *FH* mutant human tissues^23^. This subset includes peroxiredoxin-5 (PRDX5), a peroxidase responsible for mitigating cellular oxidative stress, bearing 2SC modification of C204, a residue has been shown to be necessary for protein function as mutation of this residue results in loss of peroxidase activity^27^. We further investigated functionally important cysteine residues by comparing 2SC peptides with metadata from the Uniprot knowledgebase and identified an additional five peptides where the role of the 2SC modified cysteine residue quantified on protein function has been characterized (Table 4). This target subset highlights proteins involved in regulating cellular redox states as significantly elevated in HLRCC versus NS ULMs, and include a 2SC peptide (C150) from protein microsomal glutathione S-transferase 3 (MSTG3) that is a target of post-translational modification by S-acylation^28^. We also quantify a 2SC peptide from the chloride intracellular channel protein 1 (CLIC1) protein, a chloride ion channel that regulates cellular oxidation levels and has been shown to be associated with regulating cell cycle^29^, also significantly elevated at the protein level in HLRCC versus NS ULMs (Supplemental Table 9). The modified residue (C24) has been shown to be necessary for CLIC1 protein dimerization and ion channel activity following mutagenesis analyses^30^. Lastly, we observed a 2SC peptide from creatine kinase B-type (CKB), a protein intimately involved in maintaining cellular energy homeostasis and regenerating cellular ATP from ADP^31^. The modified residue (C283) is the catalytic cysteine for CKB that has been shown to result in complete loss of enzymatic activity when mutated^32^. Although this 2SC peptide was non-significantly elevated in HLRCC ULMs, we do observe that global CKB protein is significantly decreased in HLRCC versus NS ULMs (− 0.73 LogFC, LIMMA adjusted p = 0.0002).

**Table 4:** Peptides quantified in HLRCC versus NS ULMs encoding functionally characterized 2-succinyl modified cysteine residues.

Gene name	HLRCC vs NS	P-value	HLRCC vs NS ULM	Protein name	Modified	Impact of cysteine modification	Citation
	(Median LogFC)	(MWU)	(n cases quantified)		Cysteine		(Pubmed ID)
CLIC1	2.78	< 0.0001	15_13	Chloride intracellular channel protein 1	C24	Loss of dimerization and of ion transport activity	14613939
MGST3	2.56	< 0.0001	15_13	Microsomal glutathione S-transferase 3	C150	Abolishes S-acylation; when associated with S-151	21044946
PRDX5	0.51	0.4	4_3	Peroxiredoxin-5, mitochondrial	C204	Complete loss of protein activity	10751410
NFKB1	0.479	N/A	5_1	Nuclear factor NF-kappa-B p105 subunit	C61	Suppresses S-nitrosylation-induced inhibition of DNA-binding activity	11466314
CKB	0.263	0.22	10_10	Creatine kinase B-type	C283	Complete loss of protein activity	8186255

2SC modified PSMs elevated in HLRCC versus NS ULMs, denotes total number of ULMs candidates PSMs were quantified in, n# cases quantified, as well as specific residue, impact on protein function and supporting citations (Pubmed ID).

## Discussion

This comprehensive and integrated proteogenomic analysis of ULM tissues from an exceptionally rare cohort of HLRCC patients provides a comprehensive view into the impact of *FH* mutation-driven alterations on the transcriptome and proteome in this high-risk population. These efforts extend and substantially deepen our molecular view of HLRCC ULMs and include validation of multiple transcripts previously described as altered in ULMs from HLRCC patients^20^. We further show that many of these are significantly altered at the protein level as well and further underscore that alteration of cellular glycolysis and gluconeogenesis typifies HLRCC ULMs. We show that the Nuclear factor erythroid 2-related factor 2 (NRF2) antioxidant response pathway is activated in HLRCC ULMs and that multiple NRF2 target genes are elevated at the protein level in these tumors. We also observe several targets shown to confer synthetic lethality when silenced in *FH* mutant cell lines^22^ to be altered within HLRCC vs NS ULMs including succinate dehydrogenase at the protein level in HLRCC ULMs (SDHA, 0.65 logFC, adjusted p = 7.77E−05), which catalyzes the conversion of succinate to fumarate, as well as lactate dehydrogenase. However, the functional relevance of these alterations in the context of *FH* mutant ULMs will require further investigation in disease-relevant cell line models as the synthetic lethality screen described by Boettcher et al. was performed in human embryonic kidney cells (HEK-293). Integrated analyses of HLRCC versus NS ULMs further revealed higher overall correlation of protein and transcript abundance in HLRCC versus NS ULMs. We speculate that this may be due to the higher tumor cellularity of HLRCC ULMs^33^. However, recent evidence has shown that protein and transcript abundances trend as lower in normal adjacent versus tumor tissues from > 100 lung adenocarcinoma patient tumors^14^. Although ULMs are benign neoplasms, our observation that ULMs from HLRCC exhibit significantly higher protein and transcript correlations than NS ULMs suggest HLRCC ULMs may share this characteristic with more aggressive tumors. Interestingly, we investigated correlations for > 10 K proteins and transcripts in a subset of n = 13 normal endometrial tissues described by Dou et al.^13^ and compared to n = 86 endometrioid endometrial cancers (EEC) finding correlations were comparable between these tissue types; 0.493 and 0.49 respectively. We similarly assessed n = 13 uterine serous carcinomas analyzed in this study and identified these tumors exhibited significantly higher median correlations than normal and EEC patient tumors; 0.55 (MWU P < 0.006). This would further suggest higher global protein and transcript correlations may be more characteristic of high-grade disease; 91% of EEC tumors analyzed by Dou et al. were grade 1–2. Again, although ULMs are benign neoplasms, ULMs from HLRCC patients have been described to exhibit characteristics such as morphologic atypia in comparison to NS ULMs and to further parallel morphologic characteristics of renal cell carcinoma cells, a malignant neoplasm^33^, suggesting HLRCC ULMs may exhibit more aggressive features versus NS ULMs. Our study has also identified alterations in HLRCC ULMs that have been explored in relation to therapeutic interventions for cancer treatment as well as in FH mutant backgrounds. We find thioredoxin reductase (TXNRD1) is elevated at both protein and transcript levels in HLRCC (Table 1). We further find 2SC-modified peptides from thioredoxin (TXN), a substrate of TXNRD1^34^, as elevated in HLRCC versus NS ULMs^24,25^. Arsenic trioxide targets TXNRD1^35^ and this drug has been assessed as a therapeutic strategy for the treatment of acute promyelocytic leukaemia (APL). Additionally, a recent study^36^ has shown that proteasome inhibition (marizomib) in *FH* mutant tumor cells in vitro and in vivo increases cell death by disrupting glycolysis through downregulation of ubiquitin-binding protein p62, also known as sequestosome-1 (SQSTM1) and the proto-oncogene *MYC*. We investigated these findings in our data and find SQSTM1, a downstream target gene of NRF2, to be significantly elevated at both the protein and transcripts levels (noted in results, Table 1) in HLRCC ULMs as well as *MYC* at the transcript level (+ 1.02 logFC, LIMMA adjusted p = 0.005) in HLRCC versus NS ULMS.

These data also provide insight into a post-translational modification event that results from the inactivation of *FH* activity in HLRCC characterized within clinically-relevant tissue samples, where 367 2SC-modified peptides were directly observed and quantified by MS, among which 53 have been observed in cell line models of HLRCC^26^ and 18 also described in *FH* mutant human tissues^23^ (Supplemental Table 17). We further show that proteome and transcriptome data generated for a ULM collected from an HLRCC patient (patient 1805) profiled as a NS ULM and did not harbor an *FH* mutation by WGS analyses, further exhibited very low abundance of 2SC modified peptides. These findings underscore the specificity of 2SC-modified protein targets in HLRCC tissues and the diagnostic potential of assessing this modification by immunohistochemistry^37^, or perhaps by quantifying specific 2SC modified peptides using targeted mass spectrometry approaches, such as using selected reaction monitoring (SRM) assays that could lead to prioritization of suspected HLRCC patients for genetic testing to confirm mutation in *FH*.

In conclusion, our data provides a deep proteogenomic analysis of ULMs from HLRCC patients and non-syndromic patients and further defines the impact of *FH* mutation on the proteome of disease-relevant tissues. Limitations of this study include that the majority of NS ULMs assessed were from women of AA descent while ULMs from HLRCC patients were largely from women of EA descent (Supplemental Table 1). As ULM incidence and disease prognosis is known to be racially disparate^38^, with higher incidence in AA women, interestingly we note that all HLRCC study subjects had multiple fibroids. This percentage is higher than expected based on the racial composition of the group^39–42^ and might be considered a limitation of the study; or alternately a characteristic of the HLRCC propensity to form ULMs. Such observations create an opportunity for future investigations to examine molecular alterations underlying the developmental program of ULMs correlating with patient race in the context of FH inactivation. Our study is further limited by the comparison of RNA extracted from ULM tissue sections with proteomic data generated from cryopulverized tissues which may underrepresent coordinated regulation of transcript and protein abundances impacted by intratumoral heterogeneity. Our findings underscore the implications of *FH* mutation on the HLRCC tumor proteome, highlight the dependence of these tumors on the NRF2 antioxidant response pathway and includes the description and validation of multiple transcripts and 2SC-modified protein targets that provide novel insights into molecular mechanisms underlying HLRCC disease pathogenesis.

## Materials and methods

### Uterine fibroid tissues

Flash-frozen uterine leiomyomas were obtained under an institutional-review board approved protocol IRB00093931 from Johns Hopkins University School of Medicine (Baltimore, MD). All study protocols were approved for use under institutional-review board approved protocols from Johns Hopkins University School of Medicine (IRB00093931, Baltimore, MD) and the Western IRB-approved protocol “An Integrated Molecular Analysis of Endometrial and Ovarian Cancer to Identify and Validate Clinically Informative Biomarkers” deemed exempt under US Federal regulation 45 CFR 46.102(f). All experimental protocols involving human data in this study were in accordance with the Declaration of Helsinki and informed consent was obtained from all patients. Tissues were scrolled for DNA preparation or scraped for RNA preparation from sections generated using frozen, OCT-embedded samples into microfuge tubes. Tissues were cryopulverized for proteomic sample preparation, resuspended in 100 mM triethylammonium bicarbonate (TEAB), and sonicated.

### Protein digestion, TMT labeling and offline fractionation of uterine fibroid samples

Protein was quantified by a bicinchoninic acid (BCA, Thermo Scientific) assay and 50 µg of total protein in 100 mM TEAB was transferred to MicroTubes (Pressure Biosciences, Inc) and incubated at 99 °C for 1 h. One microgram of SMART Digest Trypsin (Thermo Scientific) was added to each sample and MicroTubes were capped with MicroPestles. Pressure-assisted lysis and digestion was performed in a barocycler (2320 EXT, Pressure BioSciences, Inc) by sequentially cycling between 45 kpsi (50 s) and atmospheric pressure (10 s) for 120 cycles at 50 °C. The peptide digests were transferred to 0.5 mL microcentrifuge tubes, vacuum-dried, resuspended in 100 mM TEAB, pH 8.0 and the peptide concentration of each digest was determined by a BCA assay. Fifteen micrograms of peptide from each sample, along with a reference sample assembled by pooling equivalent amounts of peptide digests from individual patient samples, were aliquoted into a final volume of 100 µL of 100 mM TEAB and labeled with tandem-mass tag (TMT) isobaric labels (TMT10 Isobaric Label Reagent Set, Thermo Fisher Scientific) according to the manufacturer’s protocol. Each TMT sample multiplex was pooled and fractionated by basic reversed-phase liquid chromatography (bRPLC, 1260 Infinity II liquid chromatographer, Agilent) into 96 fractions through development of a linear gradient of acetonitrile (0.69%/min). Concatenated fractions (12 total per TMT multiplex) were generated for global LC–MS/MS analysis.

### RNA extraction for uterine fibroid tissue samples

Optimal cutting temperature (OCT)-embedded fresh-frozen tumor were thin sectioned, stained with H&E (including RNAseq inhibitors) and manually scraped into Buffer RLT with β-mercaptoethanol (Qiagen Sciences LLC, Germantown, MD). RNA was purified using the RNeasy Micro Kit (Qiagen) per the Purification of Total RNA from Microdissected Cryosections Protocol including on-column DNase digestion. RNA concentrations were determined using Qubit RNA HS kit (Thermo Fisher). RNA integrity numbers (RIN) were calculated using the RNA 6000 Pico Kit 2100 Bioanalyzer (Agilent).

### DNA extraction for uterine fibroid tissue samples

Tissue scrolls were generated from OCT-embedded fresh-frozen tumors or matched myometrium (n = 8 patient samples) and collected in ATL buffer (Qiagen Sciences LLC, Germantown, MD). Samples were normalized to 360 μL ATL buffer and 40 μL of proteinase K was added for lysis and incubated at 56 °C for 4 h with intermittent shaking. DNA isolation was performed according to the manufacturer’s protocol (DNA Purification from Tissues) using the QiAamp DNA Mini Kit (Qiagen Sciences LLC, Germantown, MD). DNA was eluted after a 10 min incubation with 40 μL of Buffer AE, followed by another 10 min incubation with 160 μL of nuclease-free water (Thermo Fisher Scientific) and reduced to 50 μL by vacuum centrifugation (CentriVap Concentrator, Labconco, Kansas City, MO). Quantity and purity (260/280 ratio) were established using the Nanodrop 2000 Spectrophotometer (Thermo Fisher Scientific) and Quant-iT PicoGreen dsDNA Assay Kit (Thermo Fisher Scientific) according to manufacturer’s protocols.

### Proteomics analyses and data processing

The TMT sample multiplex bRPLC fractions were resuspended in 100 mM NH_4_HCO_3_ and analyzed by LC–MS/MS employing a nanoflow LC system (EASY-nLC 1200, Thermo Fisher Scientific) coupled online with an Orbitrap Fusion Lumos MS (Thermo Fisher Scientific). In brief, each fraction (~ 500 ng total peptide) was loaded on a nanoflow HPLC system fitted with a reversed-phase trap column (Acclaim PepMap100 C18, 20 mm, nanoViper, Thermo Scientific) and a heated (50 °C) reversed-phase analytical column (Acclaim PepMap RSLC C18, 2 µm, 100 Å, 75 µm × 500 mm, nanoViper, Thermo Fisher Scientific) coupled online with the MS. Peptides were eluted by developing a linear gradient of 2% mobile phase B (95% acetonitrile, 0.1% formic acid) to 32% mobile phase B over 75 min at a constant flow rate of 250 nL/min. For both instrument platforms, the electrospray source capillary voltage and temperature were set at 1.9 kV and 275 °C, respectively. High resolution (R = 120,000 at m/z 200) broadband (m/z 400–1600) mass spectra (MS) were acquired, followed by 3 s of selection of the most intense molecular ions in each MS scan for high-energy collisional dissociation (HCD). Instrument specific parameters were set as follows for each instrument platform. Orbitrap Fusion Lumos—Full MS: AGC, 5e5; RF Lens, 30%; Max IT, 50 ms; Charge State, 2–4; Dynamic Exclusion, 10 ppm/30 s; MS2: AGC, 5e4; Max IT, 120 ms; Resolution, 50 k; Quadrupole Isolation, 0.8 m/z; Isolation Offset, 0.2 m/z; HCD, 35; First Mass, 100.

Peptide identifications and protein quantitation for TMT multiplexes were performed as recently described^43^. Briefly, .RAW data files were searched with a publicly-available, nonredundant human proteome database (Uniprot, 07/12/2016) using Mascot (v2.6.0, Matrix Science) and Proteome Discoverer (v2.2.0.388, Thermo Fisher Scientific) using the following parameters: precursor mass tolerance of 10 ppm, fragment ion tolerance of 0.05 Da, a maximum of two tryptic miscleavages, dynamic modifications for oxidation (15.9949 Da) on methionine residues, 2-succinyl (2SC) (116.0109 Da) on cysteine residues for 2SC-specific searches and TMT reporter ion tags (229.1629 Da) on peptide N-termini and lysine residues. Peptide digests for seven ULMs were included as technical replicates in several TMT multiplexes and exhibited high quantitative correlation (median Spearman Rho = 0.83 ± 0.078) with the first technical replicate was included in downstream analyses. Technical replicates for a single NS ULM sample from patient 1810 exhibited low correlation, R = 0.21, and manual inspection revealed replicate #2 was underloaded due to a quantification error, thus replicate #1 was retained for downstream analyses. Differential analyses of TMT-10 data matrixes were performed for patient samples using the LIMMA package (version 3.8)^44^ in R (version 3.5.2) of Mann–Whitney U rank sum testing in MedCalc (version 19.0.3). Significant protein and transcript alterations were visualized in heatmaps and by principle component analysis (PCA) using default settings in the ClustVis web tool^45^. Functional inference analyses were performed for significantly altered proteins using Ingenuity Pathway Analysis, and significantly enriched diseases and biofunctions predicted to be activated or inhibited were prioritized for further analyses.

### RNA-seq analyses and data processing

Sequencing libraries were prepared from 500 ng of total RNA input using the TruSeq Stranded mRNA Library Preparation Kit (Illumina) with index barcoded adapters. Sequencing library yield and concentration was determined using the Illumina/Universal Library Quantification Kit (KAPA Biosystems, Wilmington, MA, USA) on the CFX 384 real time system (BIO-RAD). Library size distribution was determined using the Fragment Analyzer TM (Advanced Analytical Technologies Inc.) with adapter dimer contamination confirmed to be less than 0.3%. Clustering and sequencing was performed on the HiSeq 500 (Illumina) using a High Output 150 cycle kit for paired-end reads of 75 bp length and an intended depth of 50 million reads per sample. Illumina bcl files were demultiplexed and converted to fastq files using bcl2fastq version 2.17. We aligned FASTQ files to hg19 by STAR aligner (2.4.2a) and quantified transcript abundance level using RSEM (1.2.22) package. Gencode v19 defined the gene models. Data were quality verified by parameters of alignment rate, base quality and GC content. Transcripts Per Kilobase Million mapped reads counts were log_2_ normalized across ULMs and z-score normalized by patient sample for correlation analyses. Differential expression analyses was performed on log_2_(TPM count + 1) transformed data using the LIMMA package (version 3.8)^44^ in R (version 3.6.0).

### DNA PCR-free library preparation and whole genome sequencing

TruSeq DNA PCR-free Library Preparation Kit (Illumina, San Diego, CA) was performed following manufacturer’s instructions. Briefly, genomic DNA (gDNA) was diluted to 20 ng/μL using Resuspension Buffer (RSB, Illumina) and 55 μL were transferred to Covaris microTubes (Covaris, Woburn, MA). The normalized gDNA was then sheared on an LE220 focused-ultrasonication system (Covaris) to achieve target peak of 450 bp with an Average Power of 81.0 W (SonoLab settings: duty factor, 18.0%; peak incident power, 45.0 watts; 200 cycles per burst; treatment duration, 60 s; water bath temperature, 5–8.5 °C). The quality of the final DNA libraries was assessed (High Sensitivity dsDNA, AATI). Per manufacturer’s protocol, library peak size was in the range of 550 to 620 bp. The DNA libraries were quantified by real- time quantitative PCR, using the KAPA SYBR FAST Library Quantification Kit (KAPA Biosystems, Boston, MA) optimized for the Roche LightCycler 480 instrument (Roche). DNA libraries were then normalized to 2 nM and clustered on the Illumina cBot 2 at 200 pM using a HiSeq X Flowcell v2 and the HiSeq X HD Paired-End Cluster Generation Kit v2. Paired-end sequencing was performed with the HiSeq X HD SBS Kit (300 cycles) on the Illumina HiSeq X. Mean genome coverage was 30X and all patient samples exceeded this expectation.

### DNA WGS processing and variant calling

WGS sample raw reads were aligned to the hg19 reference genome and further processed through the Resequencing workflow within Illumina’s HiSeq Analysis Software (HAS; Isis version 2.5.55.1311; https://support.illumina.com/sequencing/sequencing_software/hiseq-analysis-software-v2-1.html). This workflow utilizes the Isaac read aligner (iSAAC-SAAC00776.15.01.27) and variant caller (starka-2.1.4.2)^46^, the Manta structural variant caller (version 0.23.1)^47^, and the Canvas CNV caller (version 1.1.0.5)^48^. We performed focused mutational and structural variant analyses of *FH* as well as driver genes known to underlie pathogenesis of uterine leiomyomas, including *MED12, HMGA1, HMGA2, COL4A5,* and *COL4A6*^10^. We did not observe alterations for *HMGA2* that were above next generation sequence caller thresholds. Notably, we identified mutations in the *FH* gene in matched myometrium tissues, but not in companion ULMs for a subset of ULM patients (patients 1796, 1802 and 1803). Although positive evidence for mutations in *FH* within ULM tumor WGS data was present, these fell short of thresholds imposed for mutational calls and these variants were thus flagged as “LowGQX” (Supplemental Table 2). We predicted patient sample super population ancestries (Supplemental Table 1) using the methods implemented in Peddy^49^. Briefly, principal component reduction was performed on genotype calls at specific loci from 2504 samples in the 1000 Genomes project and a support vector machine (SVM) classifier was trained on the resulting first four components, using known ancestries as the training labels. Genotype calls at the same loci from each sample collected in this study were then mapped to principal component space and the trained SVM was used to predict ancestries. All classifier prediction probabilities were > 0.89.

## Supplementary Information

Supplementary Information 1.

Supplementary Information 2.

## Data Availability

Supplemental data tables include global proteome (logFC protein-abundance, Supplemental Table 5) and transcriptome (TPM level, Supplemental Table 3) level data. RAW LC–MS data files can be accessed at the ProteomeXchange Consortium using the PRIDE^50^ partner repository with the dataset identifier PXD024830Whole genome sequencing data will be deposited European Genome-phenome Archive (EGA).
