# Supplementary material for: Proteogenomic landscape of uterine leiomyomas from hereditary leiomyomatosis and renal cell cancer patients
Source: Sci Rep. 2021 Apr 30;11:9371. doi: 10.1038/s41598-021-88585-x (PMC8087684; doi:10.1038/s41598-021-88585-x)
Supplement: Supplementary file 1 — Supplementary Information 1. [file 41598_2021_88585_MOESM1_ESM.docx]

**Proteogenomic Landscape of Uterine Leiomyomas from Hereditary Leiomyomatosis and Renal Cell Cancer Patients**

Nicholas W. Bateman, PhD^1-3*^, Christopher M. Tarney, MD^1*^, Tamara Abulez, BS^1,3^, Anthony R. Soltis, PhD^4^, Ming Zhou, PhD^5^, Kelly Conrads, PhD^1,3^, Tracy Litzi, BS^1,3^, Julie Oliver, MS^1,3^, Brian Hood, PhD^1,3^, Paul Driggers, PhD^6^, Coralie Viollete, PhD^4^, Clifton Dalgard, PhD^4^, Matthew Wilkerson, PhD^4^, William Catherino, MD, PhD^7^, Chad A. Hamilton, MD^1^, Kathleen M. Darcy, PhD^1-3^, Yovanni Casablanca, MD^1,2^, Ayman Al-Hendy, MD, PhD^8^, James Segars, MD^6^, Thomas P. Conrads, PhD^1-3,5^, G. Larry Maxwell, MD^1-3,5^

^1^Gynecologic Cancer Center of Excellence, Department of Obstetrics and Gynecology, Uniformed Services University and Walter Reed National Military Medical Center, 8901 Wisconsin Avenue, Bethesda, MD 20889, USA.

^2^The John P. Murtha Cancer Center, Uniformed Services University and Walter Reed National Military Medical Center, 8901 Wisconsin Avenue, Bethesda, MD 20889, USA.

^3^Henry M. Jackson Foundation for the Advancement of Military Medicine, Inc., 6720A Rockledge Dr., Suite 100, Bethesda, MD 20817, USA.

^4^The American Genome Center, Department of Anatomy Physiology and Genetics, Collaborative Health Initiative Research Program, Uniformed Services University, 4301 Jones Bridge Road, Bethesda, MD, 20814 USA.

^5^Women’s Health Integrated Research Center, Women’s Service Line, Inova Health System, 3300 Gallows Rd. Falls Church, VA 22042, USA.

^6^Johns Hopkins University School of Medicine, Baltimore, MD, USA

^7^Department of Obstetrics and Gynecology, Uniformed Services University of the Health Sciences. Bethesda, MD, USA.

^8^University of Illinois College of Medicine, Chicago, IL 60612 USA.

*Contributed equally to this manuscript.

**Supplementary Information**

**Supplemental Table 1:** Clinical cohort details.

**Supplemental Table 2:** FH, MED12, HMGA1, COL4A5 and COL4A6 mutation and structural variant analyses.

**Supplemental Table 3:** Transcriptome matrix (TPM).

**Supplemental Table 4:** Canonical pathways enriched by proteins quantified in proteome, not transcriptome.

**Supplemental Table 5:** Global proteome matrix (median LogFC).

**Supplemental Table 6:** Spearman correlation of biological replicates; do we include these samples in the final data matrix, currently data not shown.

**Supplemental Table 7:** Spearman correlation for co-quantified proteins and transcripts

**Supplemental Table 8:** Proteins significantly altered between HLRCC and non-syndromic ULMs (LIMMA adjusted p< 0.01.

**Supplemental Table 9:** Transcripts significantly altered between HLRCC and non-syndromic ULMs.

**Supplemental Table 10:** Canonical pathways enriched by proteins and transcripts significantly altered between HLRCC and non-syndromic ULMs (LIMMA adjusted p< 0.01); 8A – Protein, 8B – Transcript

**Supplemental Table 11:** Proteins and transcripts significantly altered between HLRCC and non-syndromic ULMs (LIMMA adjusted p< 0.01)

**Supplemental Table 12:** Proteins and transcripts significantly altered between HLRCC and non-syndromic ULMs (LIMMA adjusted p< 0.01) bearing antioxidant response elements (AREs) gene promoter motifs.

**Supplemental Table 13:** Diseases and biofunctions enriched by proteins and transcripts significantly altered between HLRCC and non-syndromic ULMs (LIMMA adjusted p< 0.01).

**Supplemental Table 14:** Transcript alterations in HLRCC vs NS ULMs validated from Vanharanta et al 2006.

**Supplemental Table 15:** Proteins altered in HLRCC vs NS ULMs overlapping with synthetic lethal genes from Boettcher et al 2014.

**Supplemental Table 16:** 2SC peptide spectral match identifications.
